# Therapeutic potential and molecular mechanisms of salidroside in ischemic diseases

**DOI:** 10.3389/fphar.2022.974775

**Published:** 2022-08-19

**Authors:** Jingxuan Han, Lailiu Luo, Yicheng Wang, Shourong Wu, Vivi Kasim

**Affiliations:** ^1^ The Key Laboratory of Biorheological Science and Technology, Ministry of Education, College of Bioengineering, Chongqing University, Chongqing, China; ^2^ State and Local Joint Engineering Laboratory for Vascular Implants, Chongqing, China; ^3^ The 111 Project Laboratory of Biomechanics and Tissue Repair, College of Bioengineering, Chongqing University, Chongqing, China

**Keywords:** *Rhodiola*, salidroside, ischemic diseases, hypoxia, angiogenesis

## Abstract

*Rhodiola* is an ancient wild plant that grows in rock areas in high-altitude mountains with a widespread habitat in Asia, Europe, and America. From empirical belief to research studies, *Rhodiola* has undergone a long history of discovery, and has been used as traditional medicine in many countries and regions for treating high-altitude sickness, anoxia, resisting stress or fatigue, and for promoting longevity. Salidroside, a phenylpropanoid glycoside, is the main active component found in all species of *Rhodiola*. Salidroside could enhance cell survival and angiogenesis while suppressing oxidative stress and inflammation, and thereby has been considered a potential compound for treating ischemia and ischemic injury. In this article, we highlight the recent advances in salidroside in treating ischemic diseases, such as cerebral ischemia, ischemic heart disease, liver ischemia, ischemic acute kidney injury and lower limb ischemia. Furthermore, we also discuss the pharmacological functions and underlying molecular mechanisms. To our knowledge, this review is the first one that covers the protective effects of salidroside on different ischemia-related disease.

## Introduction

*Rhodiola* is a genus of a perennial succulent plant in the Crassulaceae family that produce flowers ranging in color from yellow to red (Galambosi et al., 2006). Having more than two hundred species in total, *Rhodiola* is widespread in high-altitude places, such as mountainous regions in Asian and European countries. Its habitat includes the southwestern part of China, India, Pakistan and North Korea, as well as Russia, Europe, especially in Alpen, and America (Khanum et al., 2005). *Rhodiola* has been used as a traditional medicine in many countries and regions for treating high-altitude sickness, anoxia, resisting stress or fatigue, and for promoting longevity (Nan et al., 2003; Chen et al., 2012; Luo et al., 2019; Zheng et al., 2019).

Previous studies have identified that *Rhodiola* has more than 140 components, with salidroside, tyrosol, rosavin, and triandrin as the main biologically active ones (Panossian et al., 2010). Among them, salidroside, categorized as phenylpropanoid glycoside with a molecular weight of 300.30, is the main active component found in all species of *Rhodiola* (Panossian et al., 2010; Qian et al., 2012; Zheng et al., 2019; Fan et al., 2020). Salidroside has been known to possess various pharmacological properties, such as resisting anoxia, anti-aging, anti-cancer, anti-inflammation, antioxidative as well as protecting the cardiovascular system (Zhang et al., 2009; Qian et al., 2012; Zheng et al., 2019; Fan et al., 2020). Accordingly, salidroside has attracted attention as a potential compound for treating ischemic diseases.

Ischemic diseases are pathological conditions caused by the obstruction of blood vessels, leading to an insufficient supply of oxygen and nutrients to organs and tissues. Ischemic diseases include ischemic stroke, myocardial infarction (MI), liver ischemia, ischemic acute kidney injury (AKI) and lower limb ischemia (Eltzschig and Eckle, 2011). Currently, standard therapeutic strategies for treating these diseases include surgery-based revascularization, organ transplantation, medication and/or application of pharmacologic agents as well as therapeutic angiogenesis (Selzner et al., 2003; Hankey, 2017). Several main components extracted from traditional herbs or plants have also been investigated for their potential for treating ischemic diseases (Han et al., 2017; Han et al., 2022). Duan et al. (2021) explored the potential of using Baicalin, the major component of the root of traditional Chinese herb *Scutellaria baicalensis*, for treating stroke; while Wang et al. (2021) examined the function of Danshen, the active component extracted from the root of *Salvia miltiorrhiza Bunge*, for treating myocardiac injury. In 2009 and 2012, Zhang et al. (2009), Chen et al. (2012) found that salidroside could ameliorate the symptoms of cardiomyopathy and hypoxia-induced neuronal damage in cardiomyocytes and mice with cortical impact injuries, respectively. Since then, more studies have revealed that salidroside possesses promising cardioprotective, anti-aging, hepatoprotective, neuroprotective and angiogenic potentials (Mao et al., 2010; Zhu et al., 2015b; Chang et al., 2016; Zhong et al., 2019). In this review, we summarize recent advances regarding the molecular mechanisms and therapeutic effects of salidroside in treating ischemic diseases, thus deepening the understanding of its therapeutic potential.

## Rhodiola and its active component—Salidroside

### Traditional use of *Rhodiola*

As a member of the Crassulaceae family, *Rhodiola* is an all-season succulent plant that grows flowers in various colors ranging from yellow to red. More than 200 species of *Rhodiola* have been found, with some of them, such as *R. rosea*, *Rhodiola quadrifida*, *Rhodiola kirilowii*, *Rhodiola crenulate,* and *Rhodiola sachalinensis*, have been studied (Panossian et al., 2010). *Rhodiola* can be found in mountainous regions with altitudes of 1,800 to 5,000 m above sea level worldwide, including the Kunlun Mountains, Himalayas, Altai Mountain and the Alps (Zhuang et al., 2019; Cunningham et al., 2020). *R. rosea*, also known as rose root, golden root, or arctic root, is the most extensively studied *Rhodiola* species, and can be found in Heilongjiang, Jilin, Tibet, Yunnan, Ningxia, Gansu, Qinghai, and Sichuan provinces in China, as well as Northern Europe, Russia, Mongolia, Korea, and Japan (Galambosi et al., 2006).

Because of its widespread habitat, *Rhodiola* has been used as a traditional medicine in many countries and regions (Nan et al., 2003; Chen et al., 2012; Luo et al., 2019; Zheng et al., 2019). In traditional Chinese medicine, *Rhodiola* has been used for increasing vital energy, invigorating blood circulation, relieving asthma, and for treating cough, diarrhea, as well as bruises (Li and Su, 2018). The Vikings used *Rhodiola* for strengthening the body during intense labor; while people in Ireland used it as a pain-healing and headache-curing plant (Panossian et al., 2010). In France, *Rhodiola* has also been used as a stimulant and astringent (Alm, 2004). Russians use *R. rosea* as a stimulant against fatigue and for treating nervous-mental diseases, neuroses and neurotic disorders; while in Siberia, people believed that consuming *Rhodiola* helped to prolong their lifespan (Panossian et al., 2010).

### Use of *rhodiola* in the modern era

The traditional use of *Rhodiola* worldwide has initiated modern scientific research regarding its pharmacological activities and active components underlying its therapeutic effects (Furmanowa et al., 1995). In the 1960’s, *Rhodiola* was extensively studied as an adaptogen, which is defined as biochemical compounds that possess adaptive effects towards stressors, such as chemical, biological and physical factors, instead of responding to them exclusively (Lan et al., 2017), and meet the following criteria: 1) not disturbing with normal conditions, 2) non-specific actions, and 3) providing normalizing actions to recover the affected conditions (Kelly, 2001; Khanum et al., 2005). *Rhodiola* could function as an adaptogen that prevents high-altitude sickness by increasing adaptation potential to high-altitude places with extreme conditions, such as low oxygen content, low temperature and increased air pressure, which affect physical conditions including blood pressure, heart rate, and tissue oxygen (Basnyat and Murdoch, 2003; Nieto Estrada et al., 2017). A human study involving 15 correspondents (9 females and 6 males) revealed that *Rhodiola* benefited the physical conditions of ski athletes, as it increased their accuracy and balance, reduced tremors and increased heart rate (Khanum et al., 2005). A preclinical study showed that *Rhodiola* could also exert a stress-resistance effect by helping *Cerevisiae* cells undergo several adaptations against stress factors in the stationary phase and increasing cell survival at the exponential phase without activating major antioxidant enzymes (Kelly, 2001; Bayliak and Lushchak, 2011).

Early studies of the pharmaceutical use of *Rhodiola* in the modern era were conducted mostly using its root extract (*Rhodiola* root extract, RRE), which is rich in secondary metabolites. RRE is usually extracted in polar solvents, such as alcohol (Wiedenfeld et al., 2007). Since the middle of the 19th century, RRE has been studied further as an adaptogen for its antioxidant properties and effects on the central nervous system (Olsson et al., 2009; Dinel et al., 2019). Studies were then extended further for different applications, such as cardiac diseases, cancer and cerebrovascular disease (Cheng et al., 2012).

**FIGURE 1:**
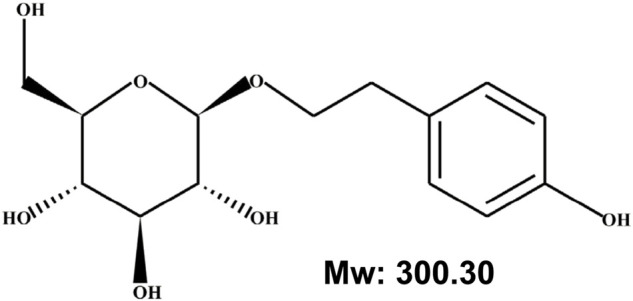
Chemical structure of salidroside.

**FIGURE 2:**
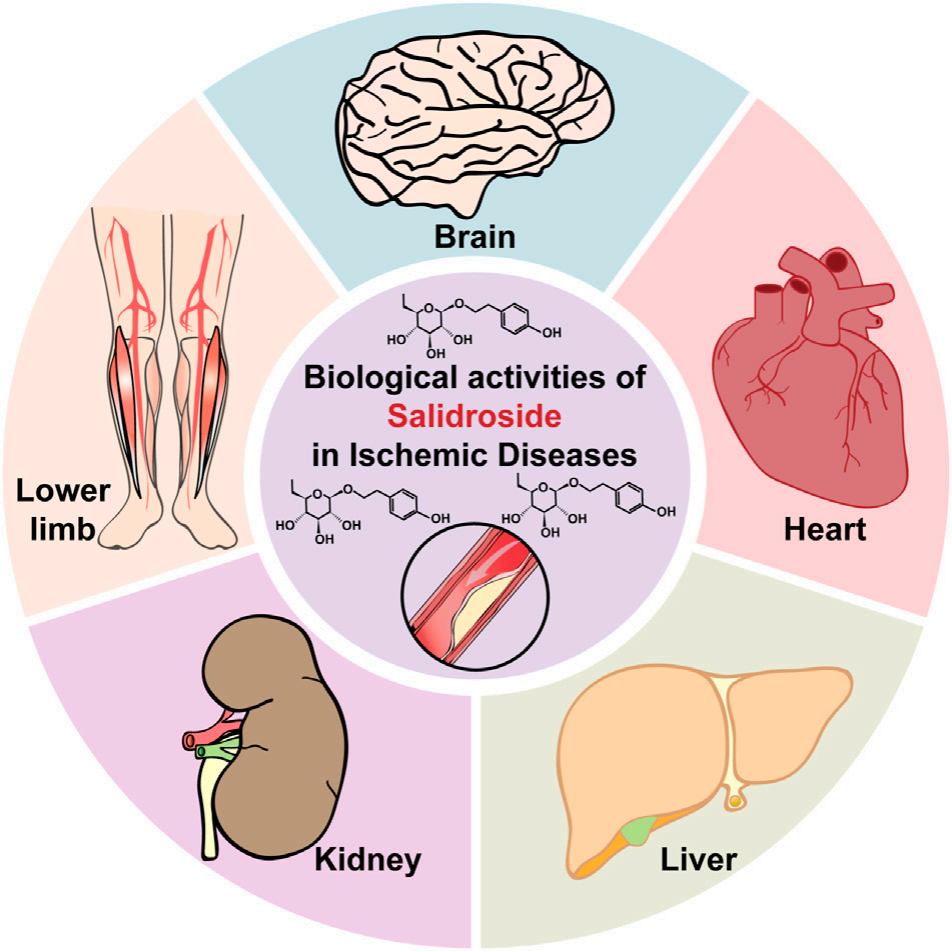
Therapeutic potential of salidroside in ischemic diseases.

The discovery of various functions of RRE subsequently drove the exploration of its active components. Since then, several secondary metabolites from RRE have been isolated, including phenylpropanoids, phenylethanol, flavonoids, monoterpenes and phenolic acids, with salidroside, tyrosol, rosavin, and triandrin as the main active components (Ming et al., 2005). Salidroside, which could be found in all *Rhodiola* species with concentrations ranging from 1.3 mg/g to 11.1 mg/g, has been known to possess various pharmacological properties (Figure 1). These include resisting anoxia, anti-aging, anti-cancer, anti-inflammation, and antioxidative as well as for protecting cardiovascular system (Zhang et al., 2009; Qian et al., 2012; Zheng et al., 2019; Fan et al., 2020). Moreover, as will be discussed below, salidroside has attracted interest as a potential drug for treating ischemic disease due to its ability to reduce oxidative stress and inflammation, while promoting cell survival and improving angiogenic potentials (Figure 2) (Mao et al., 2010; Shi et al., 2012; Xu et al., 2013; Zhu et al., 2015b; Chang et al., 2016; Cai et al., 2017; Feng et al., 2017; Zhang et al., 2018; Zhong et al., 2019).

## Ischemic diseases

### Ischemic diseases

Normal blood perfusion provides nutrition, oxygen, and metabolic compounds systematically to the whole organs, allowing them to produce and store energy. In contrast, interruptions in the blood vessels reduce blood flow and subsequently lead to an insufficient supply of nutrition and oxygen (Eltzschig and Eckle, 2011). These conditions cause severe organ and tissue damage. Furthermore, it could be lethal for cells with a high metabolism demand, such as neuron cells, cardiac cells, liver cells and renal cells, as well as cells in the hindlimbs, the most distal organ from the heart (Eltzschig and Eckle, 2011; Tymianski, 2011).

Some ischemia-manifested diseases, including stroke, lower limb ischemia and MI, can cause cell death and even mortality. Ischemic condition typically induces cell death through apoptotic, necrotic, and autophagic pathways. The insufficiency of oxygen caused by ischemia induces oxidative stress within the cells. Continuous stress leads to more reactive oxygen species (ROS) production and assemblage in mitochondria (Fu et al., 2020). ROS then affects the mitochondrial membrane permeability transition pore, causing the secretion of cytochrome c (cyt-c) which leads to cell apoptosis (Arslan et al., 2011). Ischemic condition also induces cell necrosis, as calcium ion influx, which, together with mitochondrial membrane leakage, allow extracellular fluid to enter cells, causing cell swelling and rupture (Kristian and Siesjo, 1998; Szydlowska and Tymianski, 2010). Another cellular death pathway, autophagy, is commonly due to the massive vacuolization of cytoplasm within cells (Hotchkiss et al., 2009).

**FIGURE 3:**
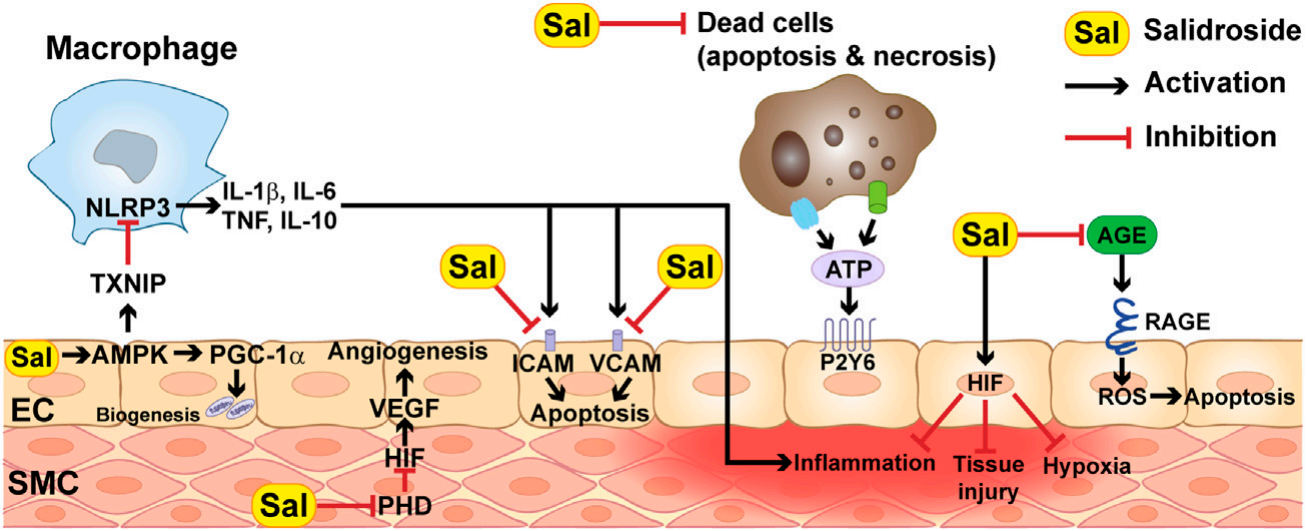
Molecular mechanisms underlying the therapeutic effect of salidroside.

On the other hand, ischemia and reperfusion also contribute to vascular dysfunction. The most common cause is through increasing vascular cells permeability and endothelial inflammation. The hypoxic state caused by less oxygen availability stimulates reduced intracellular activities of adenylate cyclase activity and the level of AMP, interrupting vascular balance permeability and leading to vascular leakage (Perrot et al., 2018). Meanwhile, dead cells that have undergone apoptosis or necrosis release ATP, which can trigger endothelial inflammation by direct binding to the NOD-like receptor family pyrin domain containing 3 (NLRP3) inflammasome and/or to endothelial inflammation receptors such as pyrimidinergic receptors (Figure 3) (Riegel et al., 2011).

### Current therapeutic strategies for ischemic diseases

Current therapeutic strategies for ischemic diseases include therapeutic angiogenesis, surgery-based revascularization, organ transplantation, medication and/or application of pharmacologic agents (Selzner et al., 2003; Hankey, 2017; Deng et al., 2020; Han et al., 2022; Tarantul and Gavrilenko, 2022). Revascularization surgery usually builds blood vessel bypasses to promote blood flow, or applies special balloons and bionic stents to expand blocked blood vessels. Another surgery-based approach is organ transplantation to replace damaged organs (Thorgersen et al., 2019). For medication treatment, drugs such as rivaroxaban and statins have been recently shown as potential drugs for treating ischemic diseases owing to their function as anti-platelet aggregation and anti-platelet thrombosis as well as for relaxing peripheral blood vessels and vasodilator (Investigators et al., 2010; Oesterle et al., 2017). Therapeutic angiogenesis, which aims to induce intrinsic angiogenic potential, promote the sprouting of new vasculature, and subsequently recover blood perfusion in the ischemic site, has emerged as one of the most promising strategies for treating ischemic diseases in the past two decades (Annex, 2013; Ko and Bandyk, 2014).

## Pharmacological studies of salidroside in ischemic diseases

### Cerebral ischemia

Cerebral ischemia is a condition where blood flow to the brain is insufficient to meet the metabolic demand, thus causing energy metabolism disorder in brain tissues. Furthermore, it might also cause oxidative stress due to excessive inflammatory cytokines production (Siesjo, 1988; Liu et al., 2011). The most well-known manifestation of cerebral ischemia is ischemic stroke, which could be caused by hypertension, atherosclerosis, smoking and complication of diabetes (Szeto et al., 2018; Ajoolabady et al., 2021; Paul and Candelario-Jalil, 2021). Ischemic stroke could lead to harmful outcomes, such as death, dementia, and disability (Ghosh et al., 2019). Decreased oxygen and glucose supply, in turn, causes insufficient metabolism and ATP production, which subsequently triggers cell death in the area of extreme ischemia. In areas with less acute ischemia, the secretion of toxic substances, such as extracellular glutamate and other excitatory neurotransmitters, leads to excitotoxicity (Iadecola and Anrather, 2011). This then drives the activation of the inflammatory response, mitochondrial dysfunction, and oxidative stress-activated programmed cell death. Therefore, therapeutic strategies for cerebral ischemia mainly aim to protect neuron functions by suppressing excitotoxicity, inflammation, apoptosis, and oxidative stress (Iadecola and Anrather, 2011).

Zhang et al. (2018) revealed that salidroside could upregulate the expressions of brain-derived neurotrophic factor (BDNF) and tyrosine kinase receptor B (TrκB), whose binding activates phosphoinositide 3-kinase (PI3K), which in turn phosphorylates phosphatidylinositol-4,5-bisphosphate (PIP2) to phosphatidylinositol-4,5,-triphosphate (PIP3). Then, PIP3 activates the mammalian target of rapamycin complex 2 (mTORC2), which, in turn, phosphorylates and activates protein kinase B (Akt) protein (Sarbassov et al., 2005). Activation of the BDNF/PI3K/Akt pathway is crucial to inhibit excitotoxicity (Dar et al., 2018). Excitotoxicity inhibition then triggers the activation of the mammalian target of rapamycin complex 1 (mTORC1), which promotes cell survival and proliferation by stimulating lipid and protein synthesis (Valvezan et al., 2017). Furthermore, animal studies with the middle cerebral artery occlusion (MCAO) mice model showed that salidroside treatment lowered ischemia/reperfusion (I/R) injury-induced neuron cell apoptosis by promoting PI3K and Akt expression levels, resulting in the recovery of mice brain post-stroke injury (Chen et al., 2012; Zhang et al., 2018; Yin et al., 2021). Moreover, salidroside could also suppress ischemia-induced apoptosis by inhibiting apoptotic proteins, such as B-cell lymphoma 2 (Bcl-2) and Bcl-2-associated protein (Bax) (Shi et al., 2012).

Microglia are the major macrophages that reside in the brain to react during brain damage (Figure 3). There are two phenotypes of microglia, M1 and M2. M1 microglia are responsible for secreting inflammatory cytokines, such as tumor necrosis factor *α* (TNF-α), interleukin-6 (IL-6) and interleukin-1β (IL-1β), while M2 microglia release anti-inflammatory cytokines, such as interleukin-10 (IL-10) and transforming growth factor-β (TGF-β) (Tan et al., 2021). Upon exposure to ischemic condition, these two microglia regulate the inflammatory response in opposite ways: M1 microglia induce inflammation and impair neurogenesis, while M2 microglia protect the brain damage by restoring the neurogenesis (Tan et al., 2021). Salidroside was found to stimulate the polarization of M1 and M2 microglia cells in the MCAO mouse model, resulting in decreased pro-inflammatory cytokines secretion from M1 cells and increased phagocytosis function of M1 cells. Moreover, salidroside also indulged in the phenotypic change of M1 cells to M2 cells, eventually increasing the number of M2 cells in the ischemic area of the brain (Liu et al., 2018). Salidroside has also been proven to reduce lipopolysaccharide (LPS)-induced microglia apoptosis by suppressing the nuclear factor kappa-light-chain-enhancer of activated B (NF-κB) and mitogen-activated protein kinase (MAPK) pathways (Hu et al., 2014).

Recruitment of T-cells and B-cells as immunosuppressor is another mechanism involved in inflammatory response triggered by cerebral ischemia. Salidroside could restore the balance of Th17/Treg cells, which is disrupted upon exposure to ischemia, thereby maintaining the peripheral neurons stability after ischemia (Yin et al., 2021).

Another mechanism of neuroprotection by salidroside is by maintaining monoamine neurotransmitters in the dopaminergic system. Monoamine oxidase (MAO) is an enzyme rich in the striatum and hypothalamus that could catalyze the oxidation of monoamines, such as dopamine, serotonin, adrenaline, non-adrenaline and histamine (Youdim et al., 2006). Dopamine plays a crucial role in preventing the delayed calcium deregulation caused by excitotoxicity (Vaarmann et al., 2013; Dar et al., 2018). Zhong *et al.* reported that salidroside administered intraperitoneally could increase the levels of MAO, dopamine, 3,4-dihydroxyphenylacetic acid, and homovanillic acid in the striatum of a focal brain ischemic injury rat model. Salidroside could also induce the expression of tyrosine hydroxylase (TH), an important enzyme in dopamine synthesis (Zhong et al., 2019).

Increased oxidative stress is a crucial factor that induces ischemic cell death. Salidroside could also ameliorate post-ischemic neuronal injury by promoting the expression of nuclear erythroid 2-related factor 2 (Nrf2), a transcription factor that binds to the antioxidant response element (ARE) and induces the expression of several antioxidant enzymes involved in ROS scavenging, such as catalase (CAT), superoxide dismutase (SOD), glutathione (GSH), and glutathione peroxidase (GSH-px). Salidroside elevated the expression of Nrf2 and protein deglycase DJ-1 (Li et al., 2019). From these downstream and upstream mechanisms, ischemic injury is ameliorated through ROS scavenging (Han et al., 2015).

### Ischemic heart disease

Blood flow in the human body is rhythmical blood distribution within the capillary beds throughout each tissue (Xu, 2020). In addition to being responsible for pumping blood, the heart itself could also suffer severe damage when the blood flow rhythm is disturbed. Ischemic heart disease (IHD) is a type of cardiovascular disease characterized by stenosis or arterial obstruction, which leads to insufficiency of blood flowing through the heart (Wong, 2014). The acute or chronic manifestations of this condition are MI, heart failure and cardiac death (Guo et al., 2018).

Salidroside could prevent the progress of myocardial ischemia from the early stage, as it could reduce exacerbation of atherosclerosis in blood vessels by inhibiting plaque formation and reducing the expression of vascular cell adhesion molecule (VCAM), intercellular adhesion molecule (ICAM), and monocyte chemoattractant protein-1 (MCP-1), which are inflammatory mediators involved in inflammatory cascade and in the pathogenesis of atherosclerosis and plaque destabilization (Figure 3) (Zhang et al., 2012).

**FIGURE 4:**
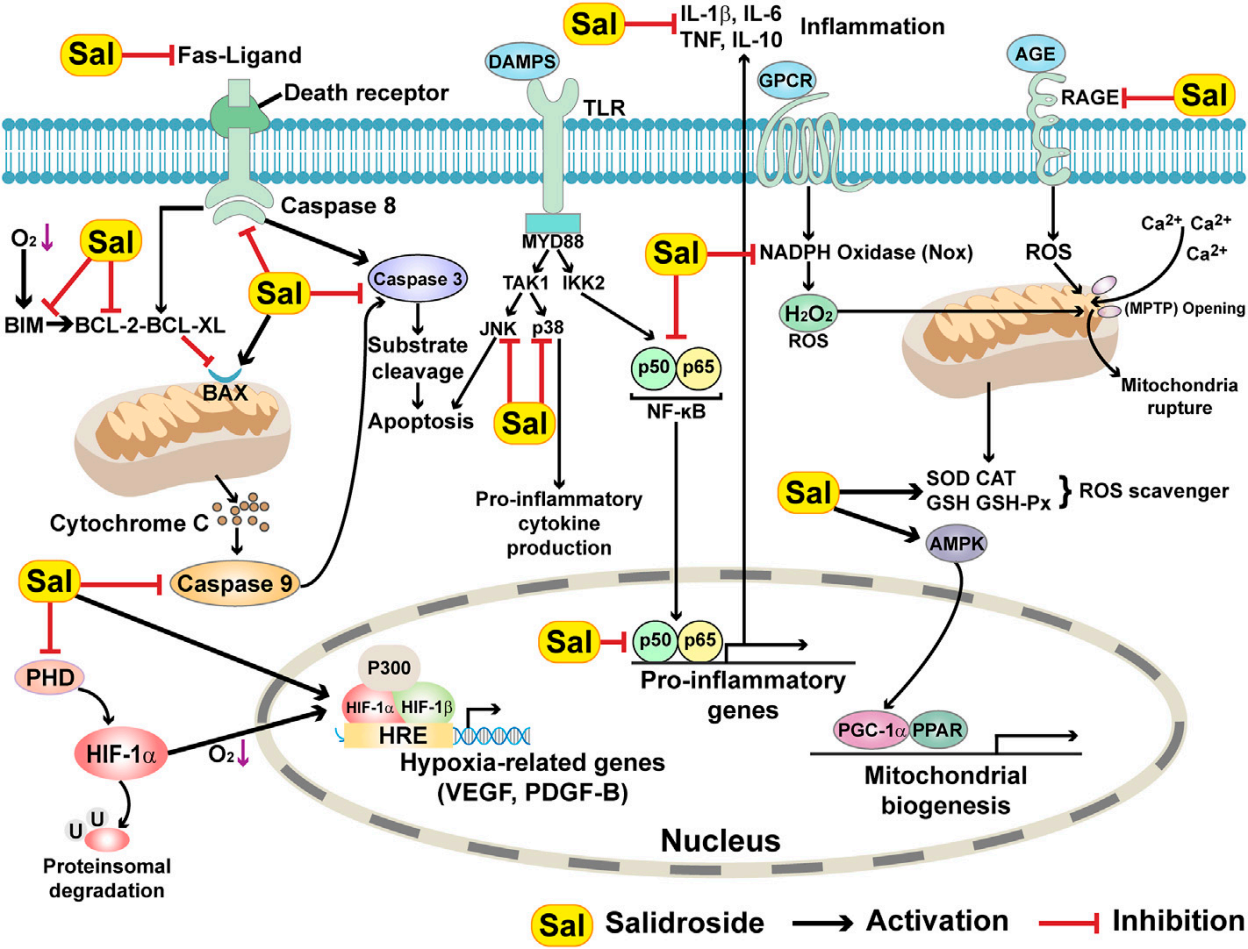
Molecular mechanisms underlying the role of salidroside in enhancing cell survival.

Ischemia can lead to cardiomyocyte cell death. Two types of apoptosis are involved in post-ischemic heart failure progression: intrinsic apoptosis, which occurs in the infarct zone within 24 h of ischemia; and extrinsic apoptosis, which occurs in the myocardium under persistent ischemic conditions (Olivetti et al., 1997; Crow et al., 2004; Ben-Shahar et al., 2021). The intrinsic apoptotic pathway, also known as the mitochondrial pathway, is also engaged in myocardium apoptosis around the infarction area. This type of apoptosis involved both pro- and anti-apoptotic proteins of the Bcl-2 family, for example, Bax and Bcl-2, respectively. Upon the occurrence of MI, Bax is highly accumulated around the infarction area, while Bcl-2 is absent in this area. In contrast, an increase in Bcl-2 could be detected in non-infarcted cardiomyocytes surrounding the infarcted cells (Misao et al., 1996; Krijnen et al., 2002). Meanwhile, in the persistent-ischemic myocardium, the death receptor, which induces an extrinsic apoptotic signaling cascade, is activated (Fan et al., 2013). For example, the death receptor Fas can bind to Fas-associated protein with the death domain (FADD), which in turn directly binds to caspase 8, one of the initial caspases, and activates it by triggering its cleavage. Activated caspase 8 then cleaves and activates caspase 3, an executor caspase, which subsequently stimulates apoptosis (Figure 4) (Hotchkiss et al., 2009). Lai et al. (2014), Li et al. (2016) *.* reported that salidroside demonstrated a cardioprotective effect by preventing cardiomyocyte apoptosis in cardiac disease mouse and rat models, respectively. Orally-administered salidroside significantly affected the expression levels of both extrinsic and intrinsic apoptotic-related factors, as it suppressed pro-apoptotic factors FasL, Fas, FADD, and Bax, respectively, while increasing the level of anti-apoptotic factors, such as Bcl-2 and Bcl-xl (Lai et al., 2014; Li et al., 2016). Furthermore, intragastric salidroside treatment in rats with I/R injury suppressed myocardial apoptosis after 30 min of left anterior descending occlusion due to an increased Bcl-2/Bax ratio and decreased caspase 3 and caspase 9 (Zhu et al., 2015b).

Myocardial ischemia could also trigger inflammation, as energy crisis and oxidative stress due to persistent I/R injury elevate the expression levels of pro-inflammatory cytokines and inflammatory transcription factors, such as NF-ĸB, IL-6, IL-1β, and TNF-α (Xie et al., 2011; Ma et al., 2015; Subramani et al., 2021). Zhu *et al.* found that subcutaneously injected salidroside could prevent autophagy in an isoproterenol (IOS)-induced myocardial injury rat model by suppressing the levels of NF-ĸB, IL-6, IL-1β, and TNF-α proteins (Zhu et al., 2015a). Moreover, activator protein 1 (AP1), a transcription factor that regulates inflammation similar to NF-ĸB, was also reduced in the salidroside-treated ISO-induced myocardial injury rat model. Meanwhile, Chen et al. (2017) demonstrated that oral administration of salidroside could also attenuate myocardial inflammation and promote angiogenesis in LPS-induced myocardial ischemia rat model.

The antioxidant potential of salidroside also plays a critical role in its cardioprotective effect. Besides suppressing ROS levels, intragastrically or subcutaneously administered salidroside could suppress the expression of non-mitochondrial ROS sources, such as NADPH oxidase 2 and NADPH oxidase 4, which are members of the NADPH oxidases (Noxs) protein family, while increasing the levels of antioxidant enzymes that could act as free radicals scavenger, such as CAT, SOD, GSH, and GSH-px in LPS-induced myocardial ischemic rat models and ISO-induced myocardial injury rat model (Zhu et al., 2015a; Chen et al., 2017).

Endoplasmic reticulum (ER) stress is also one of the complications of stress and extreme factors, such as ischemia. Unfolded protein response mediates ER stress and serves a role in the activation of three primary signaling pathways, including protein kinase RNA-like ER kinase (PERK), inositol-requiring enzyme-1α (IRE1α) and activating transcription factor 6. Salidroside mitigated hypoxia/reoxygenation injury by alleviating ER stress-induced apoptosis through declining the phosphorylation of PERK and IRE1α pathways in H9c2 cardiomyocytes (Sun M. Y. et al., 2018). Furthermore, Tian et al. (2022) found that salidroside protected against myocardial I/R by inhibiting ER stress in myocardial I/R rat model.

Not only protecting cardiomyocytes but salidroside could also benefit myocardial treatment by promoting angiogenesis *via* PI3K/Akt/mTOR pathway. Intragastrical, oral, or intracoronary administration of salidroside restored the expression of PI3K, Akt, and mTOR in myocardial ischemia in both rat and rabbit models (Xu et al., 2013; Chen et al., 2017; Chen et al., 2019). It could also promote angiogenesis by increasing HIF-1α expression and vascular endothelial growth factor (VEGF) secretion in H9c2 cells (Zhang et al., 2009), as VEGF is a growth factor transcriptionally regulated by HIF-1α and could promote the formation of the tube-like structure by endothelial cells. Furthermore, it could stimulate pericytes to express matrix metalloproteinases, leading to vasodilatation and increased permeability of the wall as well as macrophage and neutrophil chemotaxis, thereby restoring the blood vessel function and integrity (Ferrara, 2004). Oral salidroside treatment improved the hypoperfusion state in the early stages of myocardial ischemia, controlled the decline in myocardial hypertrophy and contractile force, and effectively prevented heart failure by elevating VEGF and CD34 expression levels in myocardial tissues (Chen et al., 2019).

### Liver ischemia

Unlike other organs, liver ischemia is mostly affected by external causes during surgical procedures, such as liver resection, liver transplantation and trauma (Peralta et al., 2013). However, liver ischemia can lead to fatal outcomes, such as liver dysfunction or even mortality in patients. After the injury, Kupffer cells or resident macrophages in the liver are recruited to activate the inflammatory response. These cells also release ROS and induce oxidative stress at the injured site. Furthermore, I/R injury in the liver triggers both apoptosis and necrosis in hepatocytes (Gujral et al., 2001; Guicciardi et al., 2013). Hence, targeting these types of cellular damage or death is a promising approach for treating liver ischemia injury (Konishi and Lentsch, 2017).

Several factors, such as oxidative stress, heat shock, mitogens, osmotic stress and pro-inflammatory cytokines, are activated in liver ischemia. Feng et al. (2017) found that salidroside treatment prior to liver ischemia in a rat model could attenuate liver damage. Expression levels of apoptotic factor Bax and pro-inflammatory cytokines, such as TNF-α and IL-6, were also downregulated in salidroside-pretreated rats, while that of Bcl2 was elevated. Another study conducted by the same group using concanavalin A-induced acute liver injury mice showed that intraperitoneal injection of salidroside activated PI3K/Akt signaling pathway, thus suppressing the level of pro-inflammatory cytokines as well as apoptosis- and autophagy-associated marker proteins in serum and liver tissues (Feng et al., 2018).

Non-alcoholic fatty liver disease (NAFLD) could induce liver steatosis, a major risk factor for liver ischemia. NAFLD usually implies fatty acid infiltration, inflammation, cell death and collagen deposition in liver tissues (Cotter and Rinella, 2020). Zheng et al. (2018) found that oral administration of salidroside reduced oxidative stress and alleviated NAFLD in the livers of mice with NAFLD. In addition, salidroside also downregulated the inflammatory pathway by decreasing the expression of Toll-like receptor 4 (TLR4) and NLRP3 as well as pro-inflammatory cytokines.

### Ischemic acute kidney injury

Renal I/R injury causes structural and functional damage to the renal tubules by directly inducing tubular cell death. These dying cells, in turn, trigger renal mucosal injury, tubulointerstitial nephritis and cortical fibrosis (Li et al., 2017). Thus, renal I/R injury is a major cause of AKI, which often arises from hypovolemic conditions, septic shock, surgery and transplantation. AKI can lead to chronic kidney disease and, subsequently, end-stage renal disease (Wang et al., 2014). Insufficient oxygen and nutrition supply might induce ATP depletion in kidney epithelial and endothelial cells, resulting in their cytoskeletal changes and eventually leading to their apoptosis or even necrosis (Sharfuddin and Molitoris, 2011). Furthermore, damage to endothelial cells could also worsen ischemic injury, as it contributes to hypoperfusion and eventually results in more cell death (Sharfuddin and Molitoris, 2011). In addition to cell death, similar to ischemic injury in other organs, oxidative stress and inflammation are also critical factors that could induce AKI (Ghasemzadeh et al., 2016; Nazir et al., 2017).

Sun et al. (2018b) discovered that salidroside decreased ROS levels and promoted SOD activity in human renal tubular epithelial cells (HK-2) under I/R conditions, thus promoting their viability. Salidroside also inhibited inflammation by reducing the levels of TNF-α, IL-1β and IL-6. It could also inhibit apoptosis in HK-2 cells by increasing Bcl-2 expression while decreasing Bax expression. Fan et al. (2022) established AKI septic rat models and found that salidroside injected *via* the tail vein of rats significantly reduced the plasma TNF-α, IL-1β, and IL-17A levels. Furthermore, salidroside reduced the mRNA level of Bax while increasing that of Bcl-2.

I/R injury leads to hypoxic conditions in the kidneys. The HIF protein family is a transcription factor regulated according to oxygen concentration (Semenza, 2001; Pugh and Ratcliffe, 2003). In normoxic conditions, HIF-1α protein is hydroxylated and degraded (Maxwell and Eckardt, 2016; Voit and Sankaran, 2020); however, under hypoxia, HIF-1α is stabilized and penetrates to the nucleus, where it binds to the promoters of hypoxia-activated genes, such as VEGF-A, platelet-derived growth factor B (PDGF-B), stromal cell-derived factor-1 (SDF-1), endothelial nitric oxide synthase (eNOS), erythropoietin (EPO), and heme oxygenase-1 (*HO-1*), and activates their transcription (Cao et al., 2003; Rajagopalan et al., 2007; Semenza, 2009; Zheng et al., 2012; Jain et al., 2018; Kuan et al., 2021; Nakashima et al., 2021). Zheng et al. (2012) found that salidroside treatment stimulated the accumulation of hypoxia-inducible factor-1α (HIF-1 α) protein by reducing HIF-1α protein degradation, thus promoting EPO expression in human embryonic kidney fibroblast (HEK293T).

Nephropathy is a microvascular disease in the kidney caused by endothelial cell injury and dysfunction, and is one of the major complications of diabetes (Tervaert et al., 2010; Navarro-Gonzalez et al., 2011; Flyvbjerg, 2017). Pathological conditions in diabetes contribute to ischemia, and inflammation induced by ischemia worsens diabetic nephropathy. Xie et al. (2019) showed that salidroside alleviated glomerular endothelial cell injury in diabetic nephropathy by upregulating the expression level of HIF protein. Guo et al. (2018a) found that salidroside administered via gavage inhibited proximal renal tubule cell apoptosis by suppressing Bax expression in diabetic rats undergoing uninephrectomy.

### Lower limb ischemia

Peripheral artery disease (PAD) is a pathological condition that affects a wide range of the world’s population (Fowkes et al., 2013). PAD is caused by an obstruction in blood flow, mainly by the formation of plague and/or damage to blood vessels (Creager et al., 2008). Lower limb ischemia, which is caused mainly by damage, stenosis, or blockage of the lower extremity blood vessels, is a common form of PAD. This results in insufficient blood supply to the lower extremities, which are the most distanced tissues from the heart, leaving the lower extremity in an environment of ischemia, hypoxia and nutritional deficiency (Norgren et al., 2007a; Farber and Eberhardt, 2016; Fowkes et al., 2017). Critical limb ischemia (CLI) is the most severe clinical manifestation of PAD (Criqui et al., 2021). Percutaneous revascularization or vascular surgery is the standard of immediate aid for PAD (Weinberg et al., 2011). However, revascularization approach is largely inappropriate for patients with CLI, the most severe clinical manifestation of PAD, due to severe damages in their vessels (Norgren et al., 2007b, Gerhard-Herman et al., 2017). Therapeutic angiogenesis, which aims to induce the formation of new functional blood vessels, is one of the most studied and potential therapeutic strategies to treat lower limb ischemia, including CLI patients, who are inappropriate for surgical-based therapies and are recognized as “no-option” patients (Annex, 2013).

Neoangiogenesis requires a myriad of angiogenic factors and involves different types of cells and cellular mechanisms to stimulate and promote neovascularization. PI3K can phosphorylate phosphatidylinositol (PI) to activate Akt, which in turn activates mTORC1. Activation of mTORC1 induces HIF-1α expression, which subsequently promotes angiogenesis by inducing the expression and secretion of various angiogenic factors, including VEGF and PDGF-BB (Karar and Maity, 2011).

As mentioned previously, vascular dysfunction is an outcome of an ischemic condition. However, the human body also has a homeostatic system that counteracts persistent ischemic conditions. Under ischemia, the enzymatic activity of prolyl hydroxylase domain (PHD) family to hydroxylate prolines in HIF-1α is suppressed due to the lack of oxygen as the substrate of this enzymatic reaction, thus promoting HIF-1α accumulation and allowing the body to adapt to hypoxia (Eltzschig and Carmeliet, 2011). Salidroside could be applied in therapeutic angiogenesis strategies, as Zhang et al. (2017) and Ariyanti et al., 2017 found that salidroside could act as a PHD3-specific inhibitor, thus stabilizes HIF-1α and promotes the secretory functions of skeletal muscle cells (Figure 4). This in turn elevates neovascularization through cell-cell communications between skeletal muscle cells and endothelial and/or smooth muscle cells, which are mediated by muscle-secreted multiple angiogenic factors. The skeletal muscle cell-mediated therapeutic angiogenic effect of salidroside was further confirmed in hindlimb ischemia (HLI) model mice, as intramuscular injection of salidroside enhanced neoangiogenesis and recovered blood perfusion in the HLI mice model by inhibiting PHD3, thus stabilizing HIF-1α and promoting the expression of various angiogenic factors (Ariyanti et al., 2017).

Diabetes mellitus is one of the main causes and risk factors of lower extremity ischemic disease (Cryer, 2009). Diabetes-induced systemic damage leads to lesions of blood vessels, which in turn induces the development of diabetic complications, such as diabetic lower limb ischemia and diabetic foot ulcers (Frier, 2014). Meanwhile, accumulated advanced glycation end-products (AGEs) and excessive ROS levels caused by the pathological environment of diabetes could lead to defective angiogenic potential, activation of pro-inflammatory, apoptotic and/or autophagic pathways, thus further worsening the condition of diabetic lower limb ischemia patient (Bentzon et al., 2014; Steven et al., 2017; Allahverdian et al., 2018; Grootaert et al., 2018). AGE is a covalent compound that binds to its receptor, RAGE, and is highly provoked in diabetes. Their interplays can stimulate oxidative stress by increasing NOS, causing endothelial inflammation and cell death (Figure 4). Hu et al. (2020) showed that salidroside could protect endothelial cells by suppressing the level of NF-κB, thus preventing the AGE/RAGE-stimulated NF-ĸB pathway. Furthermore, salidroside also exerts its endothelial cell protective effect by suppressing the level of NLRP3, leading to the reduction of the levels of inflammatory factors released from NLRP3 inflammasome, such as IL-6, IL-1β and TNF-β. Upregulation of AMP-activated protein kinase (AMPK) upon AGE induction is another mechanism by which salidroside protects endothelial cells (Hu et al., 2020). AMPK is crucial for endothelial cell survival, as it could exert anti-inflammatory and anti-oxidant properties by enhancing eNOS expression level through activating PI3K/Akt pathway (Zheng et al., 2019). Furthermore, it could suppress the activation of the NLRP3 inflammasome by blocking the thioredoxin-interacting protein (TXNIP).

Salidroside not only plays a direct role against ischemia but also provides synergistic effects along with stem cell treatment. Mesenchymal stem cells (MSCs) are widely used in regenerative medicine, as they are relatively easy to isolate and ideal for allogeneic transplantation without the need for immunosuppression (Hedhli et al., 2017). Moreover, MSCs can also be genetically modified to deliver specific genes required for neovascularization (Luo et al., 2019). MSCs have been used to treat ischemic diseases, such as MI and CLI, due to their wound healing and anti-inflammation properties (Gupta et al., 2017). However, poor MSCs survival in transplanted cells due to the lack of proper stem cell niches has become a hurdle for the clinical application of this strategy (Huang et al., 2019; Mohamed et al., 2020). Therefore, improving the post-transplantation survival rate is the key to MSCs’ clinical application. HO-1 is an antioxidant and a cell survival factor whose expression could be induced by several factors, including inflammatory cytokines and oxidative stress. HO-1 expression is downregulated under ischemic conditions, leading to a decrease in cell survival (Di Filippo et al., 2005). Ariyanti et al. (2019) demonstrated that salidroside pre-treatment could enhance MSC wound healing potential in diabetic mice by elevating the expression of crucial wound healing factors, such as HO-1, fibroblast growth factor-2 (FGF2), and hepatocyte growth factor (HGF). Although further investigation is needed to confirm the therapeutic effect of this combinatorial strategy in therapeutic angiogenesis, this finding indicates the potential of combining salidroside with stem cell therapy for therapeutic angiogenesis in both lower limb ischemia and CLI.

## Discussion

The utilization of *Rhodiola* has emerged from its part or whole plants in the ancient era to RRE and, further, to the use of its active component, salidroside, in the modern era (Kelly, 2001; Ming et al., 2005; Panossian et al., 2010; Bayliak and Lushchak, 2011; Wang et al., 2013; Chen et al., 2017; Zheng et al., 2019; Zhuang et al., 2019). Pharmacological research has led to the discovery of new functions of salidroside, as well as the molecular mechanisms underlying these functions. In recent years, studies have revealed the anti-aging, anti-oxidative, anti-stress, and anoxia-resisting properties of salidroside, indicating that it has a wide-range potential use both as a supplement and as a drug (Nan et al., 2003; Chen et al., 2012; Luo et al., 2019; Zheng et al., 2019). Owing to its anti-oxidative, anti-apoptotic, anti-inflammatory, angiogenic, and cell-protective properties, salidroside has been identified as a potential compound for ischemic diseases (Table 1).

**TABLE 1:** Therapeutic potential and molecular mechanisms of salidroside in animal models with ischemic diseases.

Ischemic disease	Model	Pathway	References
Cerebral ischemia	MCAO mice	PI3K/Akt/mTOR	Chen et al. (2012b), Zhang et al. (2018)
	MCAO rats	TH/MAO; Nrf2	Han et al. (2015), Zhong et al. (2019)
Ischemic heart disease	MI/RI rats	TLR4/NF-κB; Apoptosis	Zhu et al. (2015b)
	MI rabbits	PI3K/Akt	Xu et al. (2013)
	Severe sleep apnea mice	Apoptosis	Lai et al. (2014)
	AMI rats	Apoptosis	Li et al. (2016)
	MI rats	PI3K/Akt/mTOR; Nox/NF-κB/AP1	Zhu et al. (2015a), Chen et al. (2017)
	MI mice	Fas/mitochondria-dependent apoptosis; PI3K/Akt/mTOR	Chen et al. (2019)
Liver ischemia	Hepatic I/R mice	MAPK; PI3K/Akt	Feng et al. (2017), Feng et al. (2018)
	NAFLD mice	TXNIP/NLRP3	Zheng et al. (2018)
Ischemic AKI	AKI septic rats	NF-κB; apoptosis	Fan et al. (2022)
	DN rats	PHD2/HIF-1	Xie et al. (2019)
	DKD rats	Apoptosis	Guo et al. (2018a)
Lower limb ischemia	HLI mice	PHD3/HIF-1α	Ariyanti et al. (2017), Zhang et al. (2017)

However, studies regarding the use of salidroside for treating PAD were still limited at the cellular and animal models at current, and have not progressed into clinical trials yet. One of the most important obstacles might be the concentration of salidroside needed for effective treatment of ischemic disease was high, most plausibly due to the active hydrogen atoms in its phenolic hydroxyl group. Furthermore, a high dosage of salidroside increases the risk of side effects, thus impeding its clinical application. To optimize its drug-likeness, further intensive studies regarding the structure of salidroside, as well as its possible direct target in cells, which has not yet been identified, are necessary. Recently, Liu et al. (2022) synthesized more than 30 salidroside analogues and performed a structure-activity relationship study. The most optimized compound in this study could induce better neovascularization and blood perfusion recovery than salidroside in both non-diabetic and diabetic HLI mice at a significantly lower dose, suggesting the potential of applying structure-modified analogues of salidroside for clinical applications.

Several concerns need to be overcome before applying salidroside and/or its analogues for clinical use. For example, the optimal administration method, dosage, and form of the drugs might be different between patients with different types of PAD, and between patients with or without comorbid. Furthermore, preclinical and clinical systematic examinations of their side-effects as well as efficacies involving larger sample sizes are absolutely needed. Moreover, besides structural optimization as described above, combining salidroside with nanomaterials or controlled-release drug administration system could also be considered for improving its stability and efficacy.

Taken together, while further structural optimization, preclinical, and clinical studies are necessary, salidroside might become a potential drug for treating ischemic diseases.
